# An investigation into closed-loop treatment of neurological disorders based on sensing mitochondrial dysfunction

**DOI:** 10.1186/s12984-018-0349-z

**Published:** 2018-02-13

**Authors:** Scott D. Adams, Abbas Z. Kouzani, Susannah J. Tye, Kevin E. Bennet, Michael Berk

**Affiliations:** 1 School of Engineering, Deakin University, Geelong, VIC 3216 Australia; 2 Department of Psychiatry and Psychology, Mayo Clinic, Rochester, MN 55905 USA; 3 Division of Engineering, Mayo Clinic, Rochester, MN 55905 USA; 4 School of Medicine, Deakin University, Waurn Ponds, VIC 3216 Australia

**Keywords:** Closed-loop, Mitochondrial function, Oxidative stress, Deep brain stimulation, ATP sensing

## Abstract

Dynamic feedback based closed-loop medical devices offer a number of advantages for treatment of heterogeneous neurological conditions. Closed-loop devices integrate a level of neurobiological feedback, which allows for real-time adjustments to be made with the overarching aim of improving treatment efficacy and minimizing risks for adverse events. One target which has not been extensively explored as a potential feedback component in closed-loop therapies is mitochondrial function. Several neurodegenerative and psychiatric disorders including Parkinson’s disease, Major Depressive disorder and Bipolar disorder have been linked to perturbations in the mitochondrial respiratory chain. This paper investigates the potential to monitor this mitochondrial function as a method of feedback for closed-loop neuromodulation treatments. A generic model of the closed-loop treatment is developed to describe the high-level functions of any system designed to control neural function based on mitochondrial response to stimulation, simplifying comparison and future meta-analysis. This model has four key functional components including: a sensor, signal manipulator, controller and effector. Each of these components are described and several potential technologies for each are investigated. While some of these candidate technologies are quite mature, there are still technological gaps remaining. The field of closed-loop medical devices is rapidly evolving, and whilst there is a lot of interest in this area, widespread adoption has not yet been achieved due to several remaining technological hurdles. However, the significant therapeutic benefits offered by this technology mean that this will be an active area for research for years to come.

## Background

Neurological conditions are a significant global health issue, with the World Health Organization (WHO) estimating that over a billion people worldwide are effected [1]. These conditions can range from neurodegenerative diseases such as Alzheimer’s and Parkinson’s disease, to depression which is ranked as the single largest contributor to global disability (7.5% of all years lived with disability in 2015) [2]. There are a number of treatments available for these conditions with varying degrees of effectiveness, with medication and psychotherapy playing a key role as front line treatments. Unfortunately, these disorders are heterogeneous, with a lack of effective precision medicine approaches available to target the individualized pathophysiological mechanisms contributing to disease processes in any single patient, and consequent difficulty in optimizing their response to specific treatments [3, 4]. In particular, a predictable subset of patients experience complications and/or unresponsiveness to traditional treatments, even when using a combined approach with multiple interventions [5, 6]. This has led to a number of researchers investigating “closed-loop” solutions, these solutions integrate feedback on the effectiveness of the treatment allowing it to be modified “on the fly” according to each individual’s requirements [7]. However, the most appropriate targets for the feedback are still a matter of debate.

Mitochondria may play a pivotal role in several neurological disorders and may be a useful proof of concept target for a closed-loop neurological control system. [8–10]. This is not a surprise as the mitochondrion is a fundamental component of a number of crucial functions within the human body and critical for healthy brain function. A large body of evidence suggests that a disruption in normal mitochondrial function can lead to significant dysfunction in higher organisms [11]. As the brain is the most energy demanding tissue, using 20% of the body’s total energy, mitochondrial dysfunction is invariably expressed in the brain. This review explores the link between neurological conditions and mitochondrial dysfunction, and posits a generic interventional model for a closed-loop control system based on this link. In addition, this review explores several potential candidate technologies for the functional components identified in the generic model, which could be used to achieve the overall goal of a closed-loop system to treat neurological and psychiatric disorders using mitochondrial function as a biomarker.

## Mitochondria

Mitochondria are energy producing organelles found in large numbers in eukaryotic cells, comprised of four primary sections: an outer membrane, an intermembrane space, an inner membrane, and an inner matrix. Biochemical and biology studies describe mitochondria as the “powerhouse of the cell” [12]. This description is used because in most eukaryotes the common energy carrier Adenosine 5′-triphospate (ATP) is phosphorylated from ADP in the mitochondria. This mitochondrial respiration is achieved through oxidative phosphorylation, where a phosphate group is introduced into ADP to form ATP [13].

$$ \mathrm{ADP}+{\mathrm{P}}_{\mathrm{i}}+\mathrm{free}\ \mathrm{energy}\rightleftharpoons \mathrm{ATP}+{\mathrm{H}}_20 $$

This process is made possible by a proton gradient across the inner mitochondrial membrane, which describes the movement of positively charged hydrogen ions. This movement of H^+^ generates a pH gradient and an electric field (∆ψ_m_) across the inner membrane, which adds the required energy to enable the energetically unfavorable reaction between ADP and P_i_, thus producing ATP [14]. The details about this reaction were outlined in a seminal work by Peter Mitchell [15], which was recognized by a Nobel Prize in 1978.

ATP is a compound found in all living organisms, and serves as the primary “energy currency” of the cell. An ATP molecule consists of ribose, adenine, and a string of three phosphate groups, which are added or removed to transfer energy, shuttling between the triphosphate ATP, biphosphate ADP and monophosphate AMP forms, each with serially lower energy potential. Due to ATP’s high phosphate-transfer potential, it is utilized through oxidation and reduction reactions in a number of crucial biological processes including: membrane transport, cellular mitosis and muscular contraction [12]. Furthermore, ATP is a critical component in both intercellular and extracellular signaling, and acts as either a co-transmitter, or the sole transmitter in the majority of nerves in the central and peripheral nervous systems [16, 17]. Mitochondrial ATP production is an intrinsic component in a number of critical mechanisms within the human body. Hence, any disruption to this production can lead to a disturbance of many disparate functions such as cellular mitosis, muscular contraction, and neurotransmission.

In addition to ATP production, research findings indicate that mitochondria perform functions including, but not limited to the regulation of: cell apoptosis, calcium handling, free radical generation and reactive oxygen species (ROS) production [18–21]. Cellular apoptosis is a signaling cascade which leads to the death of a cell, which occurs when cells are subject to intracellular damage or physiological cues, and is commonly regulated by a subset of intracellular proteases called caspases [18, 21–23]. Mitochondria control the activation of these caspases through mitochondrial outer membrane permeabilization (MOMP), which utilizes the Bcl-2 family of proteins to regulate apoptosis [23, 24]. Calcium handling in mitochondria has been a topic of investigation since the 1960s [25]. Current evidence suggests that calcium levels within the mitochondria not only play critical intracellular signaling roles, but assist in determining thresholds for cellular death, and play an essential role in fine-tuning cellular energetics [26, 27]. However, even after more than five decades of investigation, there still remain a number of significant open questions in this domain, particularly in the specific physiological roles of mitochondrial calcium handling [27]. The multifaceted role of mitochondria suggests that even subtle dysfunction could have far-reaching consequences.

## Linking mitochondrial dysfunction and neurological disorders

The links between mitochondrial dysfunction and neurological disorders have been an active area of investigation. Tissue in areas with high energy demands, such as the brain, heart and endocrine system is highly reliant on mitochondrial based aerobic respiration [28]. The brain is highly dependent on a continuous and efficient energy supply and is consequently considerably vulnerable to mitochondrial perturbation. Evidence suggests that this perturbation may significantly impact neural viability, neural plasticity and long-term cellular resilience, which are linked to the pathogenesis and pathophysiology of several neurological and psychiatric disorders [29–34]. This paper examines some most common conditions including Parkinson’s disease, Alzheimer’s disease and bipolar disorder and explores their links to mitochondrial dysfunction.

### Alzheimer’s disease

Alzheimer’s disease (AD) is a progressive neurodegenerative disease first described in the early 1900s by Alois Alzheimer which is estimated to affect 6% of the population over 65, with the incidence rate increasing exponentially with age [35–37]. AD is characterized by progressive decline of cognitive function usually beginning with difficulties with recent memory but eventually affecting all intellectual functions [38]. The majority of AD cases are sporadic, for which the causal factors are still unknown [39]. The prevalent model of AD (the Amyloid Cascade model) propounds that AD pathogenesis is linked to the overproduction of the beta-amyloid (Aβ) peptide in neurons, however, the exact cause of this overproduction is disputed [40, 41].

In 2004, Swerdlow RH and Khan SM [42] posited a “mitochondrial cascade hypothesis” as a specific cause of sporadic late-onset AD. This hypothesis provides a unifying framework for AD pathology, citing low rates of oxidative phosphorylation, and high rates of ROS production as a key cause for sporadic, late-onset AD [42–44]. This hypothesis explains one of the key characteristics of AD, which is the correlation of incidence with increasing age. There is a significant amount of evidence suggesting that over time, mitochondrial DNA accumulates an increasing number of mutations resulting in a marked decrease of ATP production, concurrent with increased ROS generation [45–48]. A number of researchers have identified this mitochondrial dysfunction as a potential pathway for developing more efficacious intervention strategies [41, 49]. However, this link between AD, mitochondrial dysfunction and Aβ overproduction is an area of intense current research, and the development of effective tools and techniques to aid in this research is of considerable importance [41, 50–54].

### Parkinson’s disease

Parkinson’s disease (PD) is the second most common neurodegenerative condition after Alzheimer’s disease, characterized by progressively increasing resting tremor, rigidity, bradykinesia, and postural instability [55–57]. The connection between this disorder and mitochondrial dysfunction has been investigated since the early 1980s, when a number of drug users developed parkinsonism after mistakenly administering a synthetic drug which inhibited complex I, an enzyme crucial to the mitochondrial respiratory chain [58, 59]. Since this time, a compelling amount of evidence has been discovered indicating that PD is associated with mitochondrial dysfunction, and has identified this dysfunction as a potential pathway for intervention [60–66]. Mitochondrial dysfunction in this case can be expressed as a decrease in ATP production and an increase in ROS generation [59, 64, 65, 67]. One of the difficulties in studying mitochondrial response is the specificity required of the assaying technique. A study by Schapira AH, Cooper JM, Dexter D, et al. [68] found that in postmortem examination, PD tissues exhibited a 30–40% inhibition of Complex I activity. However, Davey GP and Clark JB [69] found in a whole brain assay that Complex I activity could be reduced by ~ 72% before significant changes in mitochondrial respiration took place [59, 67]. Not until a further study, specifically investigating synaptic mitochondria, was found that as little as a ~ 25% reduction in Complex I severely inhibited ATP production [70]. This clearly demonstrates the need for high accuracy measurement techniques to be able to detect potential dysfunction, which may only be present in a specific region, particular to that disorder. There is a great deal of complexity in studying a disorder with a heterogeneous pathogenesis and pathophysiology, and further research is still required to develop a more complete understanding of the mitochondrial link in PD.

### Amyotrophic lateral sclerosis (ALS)

Amyotrophic lateral sclerosis (ALS) or Lou Gehrig’s disease is a fatal neurological disease with a life expectancy of approximately 3 years after onset [71]. The pathogenesis of ALS is still not fully understood which significantly limits the treatment options available [72]. Some of the mechanisms which have shown links to the condition include oxidative stress, protein misfolding and endoplasmic reticulum stress, which are intrinsically connected to mitochondrial dysfunction [73–77]. As with PD and AD, it has been suggested that ALS shares a link with significant disruption to the mitochondrial respiratory chain and a decrease in ATP production and an increase in ROS generation, hence, addressing this through antioxidants and mitochondrial modulators has been a target for a number of treatments [72, 78–81]. Unfortunately, as seen in a meta-analysis by Benatar M [82], these techniques have had difficulties transitioning from the lab to a clinical outcome. Cozzolino M and Carrì MT [72] suggested that the investigated treatments may be too narrow to show clinical significance, and that more complex treatment options directed at multiple therapeutic targets should be investigated.

### Major depression and bipolar disorder

Depressive disorders are a significant cause of disability globally, and have consistently been among the leading causes of burden in the Global Burden of Disease (GBD) studies [83, 84]. The exact pathophysiology of unipolar depressive disorders such as major depressive disorder (MDD) is not comprehensively understood, and depression lacks a specific reproducible biomarker [85, 86]. This in part is likely due to the heterogeneous nature of the illness. New theories have been proposed in light of recent research demonstrating several biological factors contributing to the pathophysiology of depression and other mood disorders [86–88]. One of the first large scale studies to show a relationship between genetic abnormalities and MDD was a study by the CONVERGE consortium [88] on more than 10,000 subjects with recurrent MDD. This study identified two loci which contribute to the risk for MDD, including the SIRT1 gene which protects against oxidative stress through regulating mitochondrial function. In addition, several studies on the co-morbidity of mitochondrial disorders and MDD have shown that individuals with abnormal cerebral energy metabolism (ATP production) have a much higher incidence rate of MDD and bipolar disorder than the general population [10, 89–91]. This does not, however, provide a generalized pathogenesis for MDD, as the disorder has a highly complex psychiatric phenotype without a unitary pathogenesis or pathophysiology [85]. What this does elucidate, however, is that investigation into treatments in this area could potentially be valuable for elucidating critical operative pathways, which may translate into benefits for sufferers, potentially including the subset of patients who are resistant to traditional anti-depressant intervention [87].

In comparison, the links between bipolar disorder (BPD) and mitochondrial dysfunction are much clearer, with a growing body of evidence indicating that mitochondrial respiratory chain perturbations are closely linked to the pathogenesis and progression of the disorder [8, 34, 92–95]. BPD is a biphasic disorder where patients exhibit distinct manic and depressive stages. A growing body of evidence is beginning to associate the mania phase of the disorder with an increase in cerebral energy generation (ATP production) and resting energy expenditure, whereas depression is associated with the inverse [96–98].

A recent review of the literature by Morris G, Walder K, McGee SL, et al. [97] presents a model of BPD which brings together the results of many separate studies, and expounds upon links implicating state dependent mitochondrial dysfunction in the biphasic pathophysiology of BPD. Several studies have found that parameters directly linked to ATP production, including uric acid and intracellular calcium, are increased in patients experiencing the manic phase of the disorder, and decreased in patients experiencing depression, these changes can alter week to week [8, 99–101]. This shows the potential of mitochondrial dysfunction for more than just a long-term indicator of progression, but as a dynamic biomarker which may provide information on dynamically changing symptoms.

A common intervention for BPD is mood stabilizing drugs such as lithium and valproate, which have effects on both the manic and depressive stages of the disorder. The work of Cui J, Shao L, Young LT, et al. [102] has shown that these agents also have multiple actions on mitochondrial biogenesis. Results from the Systematic Treatment Enhancement Program for Bipolar Disorder (STEP-BD) study in the USA found that 40% of patients with bipolar disorder used 3+ medications and 18% used 4 or more, driven by suboptimal outcomes with first line therapies [103]. The high prevalence of utilization of complex drug regimens illuminates one of the key issues in managing a multi-phase disorder such as BPD. If a treatment option was available which could detect the pathophysiology underpinning the current phase of the disorder and applied an appropriate intervention, this could greatly increase the efficacy of BPD treatment.

## Generic model

**Fig. 1:**
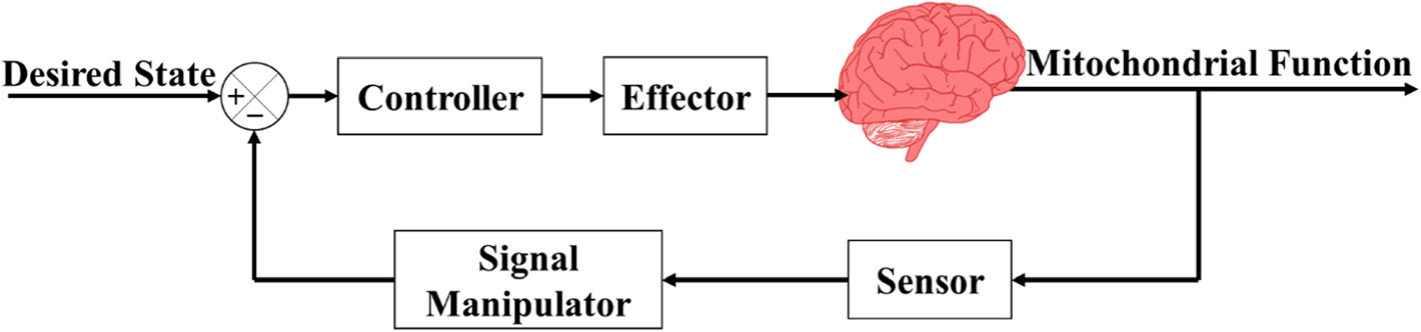
Generic model of a mitochondrial dysfunction based control system

In response to the issues outlined in Section III, the authors propose a generic model of an in-vivo closed-loop, feedback based control system for neurological conditions, based on mitochondrial function, shown in Fig. 1. This model aims to describe the high-level functions of a system designed to control neurological conditions, grounded on feedback-based modulation of mitochondrial function, simplifying comparison and enabling future meta-analysis. Open-loop treatment options provide the same intervention to the patient irrespective of system status or response. A closed-loop system through the inclusion of a feedback component enables the system to dynamically react to the biologic effect and the subsequent treatment needs of a patient.

The key components of a system aiming to control these conditions in-vivo are the sensor, signal manipulator controller, and the effector. The sensor component allows for the current mitochondrial function of the subject to be measured, this may be a direct measurement or an indirect measurement, and communicated to the control system. The sensing component comprises of a single transducer or multiple transducers; these transducers can vary in location as well as type. The signal manipulator element receives the signal from the sensor and alters it such that it can be read by the control system. The controller component receives the output from the signal manipulation element and estimates the mitochondrial function. Once this is complete, the controller runs through a predetermined control algorithm and determines what signal to send to the effector. The effector is used to alter the conditions within the in-vivo tissue. The effector component comprises one or more methods which can be instigated in a controllable manner.

## Sensor

The key aim of the sensing component is to accurately quantify mitochondrial function or a related function in-vivo and pass this measurement to the control system. Through the investigation carried out in Section III, it was observed that all the examined disorders expressed some common features during mitochondrial dysfunction: abnormalities in intracellular calcium regulation, altered ATP production, and increased oxidative stress leading to apoptosis (programmed cell death). ATP levels in the extracellular matrix have been closely linked to mitochondrial dysfunction based apoptosis, as well as intracellular calcium regulation [104–108]. The mechanism of purinergic receptor activation (particularly P2X7R) by extracellular ATP has been recognized as a danger signal within the brain. This is linked directly to alterations in intracellular calcium levels and mitochondrial function, and can significantly alter the mitochondrial membrane potential [105, 107–110]. During the activation of these receptors, the concentration of extracellular ATP can increase 10^6^ from the background of ~ 10 nM, up to 1 mM [104, 111].

In order for any ATP sensing mechanism to be effective in this context, it must be selective, sensitive, stable, and fast to respond. Additionally, the sensing technique must also be suitable for long term in-vivo use in unconstrained subjects. Ideally this technique would be non-invasive, however the great difficulty in accurately sensing neurological function in real-time, means that the accuracy of such a technique is significantly reduced. There are a number of neurological targets which show clear potential for sensing mitochondrial dysfunction in this manner, however, these would be selected depending on the disorder. The substantia nigra (a nucleus in the midbrain) and the cholinergic nucleus basalis, are an obvious target for PD as a number of studies have linked mitochondrial dysfunction in these regions with the disorder, while for AD the locus coeruleus may be a more effective target, in mood disorders the changes appear to be more global [67, 68, 112].

Several techniques are available which can be used to sense extracellular ATP function in-vivo. They use a variety of transduction mechanisms to convert the ATP concentration to a measurable quantity. One of the earliest studies describing a method of quantifying ATP concentration was published by Kerr SE and Daoud L [113] in 1935. This study detected the number of phosphates in a molecule in 24 species of animal by measuring the quantity of hydrolyzable phosphorus. However, a 1947 discovery by William McElroy uncovered that ground fireflies emitted a flash of light when combined with ATP [114]. This occurs because firefly luciferin when combined with ATP and magnesium produces luciferase with an intensity directly related to the ATP concentration within the sample [115]. The discovery of a method which reliably and accurately allows for the calculation of ATP within a sample opened up a new era of chemiluminescent assaying for quantifying ATP concentrations.

Adams S, Kouzani AZ, Bennet K, et al. [116] found that modern chemiluminescent ATP transduction techniques were highly accurate and were able to retrieve results rapidly. Many research studies have successfully used these techniques to measure the concentration of ATP in-vivo, however, all the works reviewed as part of this study required the subject to be stationary, and gathered results using highly sophisticated imaging equipment which is resistant to miniaturization [117–122]. Traditional chemiluminescent techniques require analysis through vision recognition techniques which is computationally intensive, and requires a powerful control system to be effective [123]. More modern techniques utilize optical fibers in order to gather assays in a more selective manner, allowing for a higher special resolution; however, these optic fibers often have diameters in the hundreds of microns and require complex readers in order to gather results [124]. In addition to these disadvantages, much of the surveyed research which used chemiluminescent techniques to measure cerebral ATP was performed on postmortem subjects or on in-vitro cell cultures [125–127].

In order to address some of the drawbacks found in chemiluminescent sensor systems, a number of researchers have been investigating electrochemical techniques such as amperometric biosensing as an alternative. Constant potential amperometry consists of the application of a fixed potential between a working electrode and a reference electrode, while measuring the resultant current. The current is a result of oxidation or reduction of analytes on the surface of the working electrode, and is proportional to the concentration of the analyte in the media [128]. This technique allows for measurements to be taken with high spatial and temporal resolution, while remaining suitable for miniaturization [129, 130]. When using unmodified electrodes, this technique often does not achieve a high degree of selectivity, so films or enzymes are added to the electrodes to increase the electrochemical response of the analyte of interest, on the working electrode surface [130]. Amperometric biosensors are electrodes which are integrated with biological recognition layers to allow for a large variety of analytes to be measured (including ATP) with a high degree of sensitivity and selectivity [131]. This type of sensor is suitable for miniaturization, and can even perform measurements while being carried by the subject during normal activity [132].

As can be seen in Table 1, there has been significant research efforts into developing a biosensor to accurately determine ATP concentration. One important aspect of these studies is that none measure ATP directly, but measure the oxidation of related compounds which are related to ATP concentration such as hydrogen peroxide [133]. The reason for this is that ATP is electrochemically active at ~ 1 V which is an unsafe level of voltage to be continuously applied [134]. Also, it can be seen that these sensors react relatively slowly, with none of the sensor designs producing a result in less than 10s. This means that any control system connected to these sensors would have a significant delay in the received readings. The short active lifetime of the sensors is an issue, due to the use of enzymes associated with the detection mechanism. As was noted in the study by Kueng A, Kranz C and Mizaikoff B [133], a response time of under 100 ms would be ideal for measuring and quantifying ATP at the cellular level. A key advantage of this approach is that highly skilled personnel and sophisticated computing resources are not required to interpret the results as in chemiluminescence. This makes amperometric biosensing a more viable proposition than chemiluminescence as the sensing component in a closed-loop control system.

**Table 1:** Comparison of amperometric biosensors for measuring ATP concentration

Title	Recognition Layer	Measured Quantity	Sensitivity (pA/μM)	Response Time	Electrode Size (μm)
Microelectrode Biosensor for Real-Time Measurement of ATP in Biological Tissue [153]	Glycerol kinase and glycerol-3-phosphate oxidase	H_2_O_2_	250	10s	25
ATP microelectrode biosensor for stable long-term in vitro monitoring from gastrointestinal tissue [199]	Glucose oxidase and hexokinase	H_2_O_2_	45.8	40–50s	50
Developmental aspects of amperometric ATP biosensors based on entrapped enzymes [200]	Pyrroloquinoline quinone-dependent glucose dehydrogenase and hexokinase	Various	290	50–200 s	25
Co-immobilization of glucose oxidase and hexokinase on silicate hybrid sol–gel membrane for glucose and ATP detections [201]	Glucose oxidase and hexokinase modified with silica hybrid sol-gel film	H_2_O_2_	10.8	15 s	3 × 10^3^
Poly(benzoxazine) as immobilization matrix or miniaturized ATP and glucose biosensors [202]	Glucose oxidase and hexokinase with entrapment from a poly(benzoxazine) derivative	H_2_O_2_	48.5	–	25

**Fig. 2:**
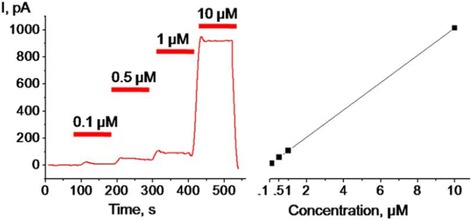
Response of an amperometric glycerol kinase and glycerol-3-phosphate oxidase biosensor to different concentrations of ATP in solution. The linear relationship between output current and ATP concentration can be clearly observed (Adapted from: Palygin O, Levchenko V, Ilatovskaya DV, et al. [135])

The responses of an amperometric biosensor to ATP concentration can be seen in Fig. 2. As can be observed from the presented results, the sensor takes a number of seconds to stabilize after the addition of ATP [135]. This makes the method unsuitable for tracking highly dynamic changes in ATP concentration. In situations where amperometric sensing is unsuitable, or unable to gather the results required, other related electrochemical techniques are available. One such technique is cyclic voltammetry. Cyclic voltammetry is an electrochemical technique where the potential is increased and decreased in a repeated manner, allowing for identification and measurement of the analyte from its electrochemical oxidation/reduction at known potentials [136–138]. A subtype of cyclic voltammetry called Fast Scan Cyclic Voltammetry (FSCV) is of particular interest when examining ATP concentrations as it allows for readings to be taken in the millisecond range [139]. FSCV varies the voltage potential at a high speed, often at more than 1 kV/s with a cycle speed over 10 kHz in order to gather highly transient readings from the analyte [137, 140]. An additional advantage of this high speed changing of potential is that ATP can be directly measured, as the electrodes do not maintain a high potential for an extended period of time. FSCV is a well-established electrochemical assaying technique, and has been used on in-vivo brain tissue to gather sub-second readings of both dopamine and adenosine [137, 141–143].

Recently a number of studies have investigated the potential of FSCV to measure ATP concentration in-vivo [144, 145]. This technique has several advantages over the more established amperometric biosensors. It is designed to take high speed measurements in the millisecond range, and the electrodes used do not require enzyme coatings and can thus be significantly smaller (on the order of 7 μm diameter by 50 to 100 μm long) [137, 142]. Smaller electrodes are preferred as they cause less damage when being implanted into brain tissue [146]. A number of research studies have identified these advantages, and investigated FSCV for use as the sensor portion in a closed-loop control system. de Araujo CE, Abatti PJ, Da Cunha C, et al. [147], Chang S-Y, Kimble CJ, Kim I, et al. [148], and Bozorgzadeh B, Schuweiler DR, Bobak MJ, et al. [149] all describe closed-loop control systems which use FSCV as the sensing element. However, in all of these cases the sensing target is dopamine release. If these techniques were instead modified to detect and sense abnormalities in extracellular ATP, it could open up treatment options beyond those which are currently possible.

## Signal manipulator

The second part of the generic model depicted in Fig. 1 is the signal manipulator element. This component receives the output of the sensor and transforms it into a signal which can be read by the control system. There are multiple methods which chemiluminescent sensors use to transform luminescence into useful signals depending on the type of sensor being used. Traditionally, chemiluminescent assays are performed using an imaging chamber, super cooled CCD camera (for noise reduction) and a display, when used for manual interpretation, or image based signal processors for automatic analysis [126, 150]. Unfortunately, this means that this technique requires significant computing resources in order to convert the signal into one decipherable by the control system, additionally the equipment required is large, and resistant to miniaturization.

Highly sensitive chemiluminescent ATP sensors have been developed by Iinuma M, Ushio Y, Kuroda A, et al. [124] which immobilize luciferase molecules at the end of an optic fiber to increase the spatial resolution and increase the portability of the sensing element. However, even this type of sensor requires sophisticated equipment (photon detectors), resistant to miniaturization, in order to convert the results to a format utilizable by a control system [124, 151].

In comparison to the complexity of the chemiluminescent techniques, amperometric biosensors and FSCV based sensors have relatively simple output signals for a control system to read as the output is an analogue electronic current which can be input to a control system through a transimpedance amplifier and an Analogue to Digital Converter (ADC) [129]. However, as the pertinent signals are frequently in the nano-ampere range, signal manipulation is required in order to produce a measurable signal. This is normally achieved through amplification circuitry which aims to increase the signal amplitude without adding a significant amount of noise to the measurement [152]. This conversion and manipulation is usually performed by commercially available data acquisition modules attached to a computer [153–156]. However, a number of recent investigators have developed their own discrete circuitry to perform this task for size reduction and energy efficiency in order to connect to a custom microcontroller based control system and ultimately create an implantable system.

## Controller

Once the signal from the sensors has been converted into an appropriate format, the controller utilizes the state of the system, and calculates what action (if any) is appropriate to take. The lack of research on the topic of closed-loop control systems sensing mitochondrial function was notable, and to the best of our knowledge, this idea has so far not been considered in the existing literature. Several studies have, however, investigated the possibility of amperometric biosensing and FSCV based control systems for neurological conditions, using targets other than mitochondrial function.

de Araujo CE, Abatti PJ, Da Cunha C, et al. [147] described a FSCV based system which allows for control over dopamine concentration in-vitro. This system uses a number of Arduino-based prototyping boards as the hardware for the FSCV signal production and measurement acquisition while using LabView (National Instruments) on a host-computer for the controller. The effector used in this study was a syringe pump which increased the amount of dopamine in the system. The control algorithm used in this system is not explicitly stated, however, the authors do mention that the control is able to control the analyte concentration to within +/− 0.8 μmol/L range with an oscillation period of approximately 80s, indicating the system used is likely some form of PID control [147]. The key disadvantage of this system is that it requires a host-computer to operate, and the control is operated through the Lab-View software which severely limits the potential for use of this system in-vivo.

**Fig. 3:**
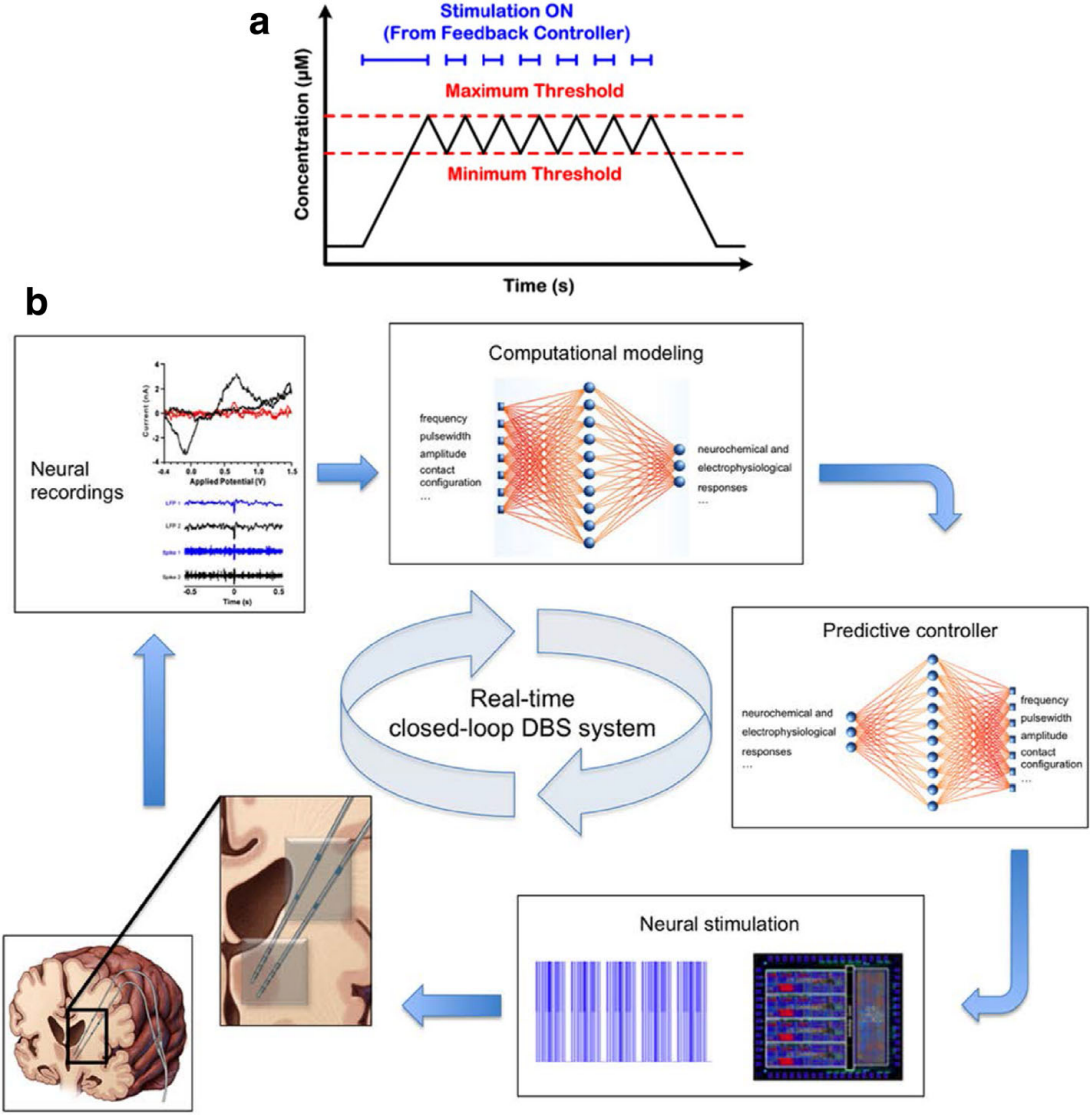
**a** Diagram showing the “on-off keying” control scheme used by Bozorgzadeh B, Schuweiler DR, Bobak MJ, et al. [149] as part of their control system. Adapted from: Bozorgzadeh B, Schuweiler DR, Bobak MJ, et al. [149]. **b** Model of the real-time closed-loop system developed by researchers at the Mayo Clinic in order to control dopamine response within the brain. Adapted from: Grahn PJ, Mallory GW, Khurram OU, et al. [157]

Other researchers have developed systems which avoid these issues, one such system is the “Neurochemostat” developed by Bozorgzadeh B, Schuweiler DR, Bobak MJ, et al. [149]. This system is a single CMOS chip design, utilizing FSCV as the sensing element, and deep brain stimulation (DBS) as the effector. This device is highly suitable for in-vivo use, and the results of initial in-vivo testing were included as part of the study. The control scheme used was termed “on-off keying”, where the effector was activated to an “On” state when the sensor detected a value above a “High” threshold value. The stimulation was deactivated when the sensor reported a value below a “Low” value [149], this is illustrated in Fig. 3 (a).

This system is suited to long-term in-vivo operation on freely moving subjects, as it is entirely self-contained and thus, no external devices are required in order for the control system to operate. However, the control algorithm used is a relatively simple one, and there is a great deal of room for further investigation into more effective control schemes. A much more comprehensive approach was that taken by the Mayo Clinic reported in the work by Grahn PJ, Mallory GW, Khurram OU, et al. [157] seen in Fig. 3 (b). A two-layer adaptive neural network was used to develop a system model, and a predictive controller was employed to achieve control of dopamine release within the brain of the subject. This solution is highly suitable for use in-vivo, and the results were verified through testing on a number of in-vivo models. The disadvantage of this approach is that it is computationally intensive and requires much more sophisticated hardware than the device described by Bozorgzadeh B, Schuweiler DR, Bobak MJ, et al. [149] in order to be run in real-time.

Viewing the state of the current research in this area, there is clearly a number of aspects which are ripe for further investigation. There is a great deal of room between the simplicity of the control approach taken by Bozorgzadeh B, Schuweiler DR, Bobak MJ, et al. [149] and the complexity of the approach taken by Grahn PJ, Mallory GW, Khurram OU, et al. [157]. This review did not find any publication investigating the efficacy of control schemes for this application in either a quantitative or qualitative fashion. This could reflect the lack of a standardized, commercially available platform which would allow researchers to develop and compare available methods of control.

## Effector

The final part of the system described in the generic model in Fig. 1 is the effector. The role of the effector in this model is an output which alters the mitochondrial function, within the environment being sensed. The effector of this type of system can take many forms such as deep brain stimulation, drug therapy, or even a cognitive or sensory task or stimulus, however, only those that can be operated by an electronic control system will be considered here.

### Deep brain stimulation

The first effector method to be considered is deep brain stimulation (DBS) which has been used in neurological control systems studies. The stimulation parameters of a DBS system are highly customizable and can be altered through an electronic control system. This treatment has established effectiveness in a significant number of PD cases [149, 157–160]. DBS is an invasive neuromodulatory approach, where electrodes are implanted into the brain, and electrical impulses are delivered to the brain tissue in order to alter the neurological signaling within the target area [6]. DBS has been used as an intervention for PD since the late 1980s. DBS is currently used extensively, however, the exact mechanisms behind its efficacy as a treatment are still not completely understood [161, 162].

Following its success as a treatment in PD, DBS has also been investigated as a potential treatment for Alzheimer’s disease with some preliminary studies finding that in appropriate patients, DBS might delay cognitive decline, and result in improved quality of life [163–165]. Treatment resistant depression (unipolar and bipolar) has also been identified as a potential target for DBS therapy, with several studies finding significant improvement in depressive symptoms from patients receiving the treatment [166–168].

In addition to assisting with other aspects of the disorders, several studies have shown that electrical stimulation can also have a direct impact on mitochondrial function, [169–171]. A study by Bekar L, Libionka W, Tian G-F, et al. [172] found that the effectiveness of deep brain stimulation could be closely linked to the release of extracellular ATP. Their research indicated that the activation of the Adenosine A1 receptor reduced both tremor and side effects commonly associated with DBS treatments. If a system was designed which worked in concert with this finding, the existing DBS treatments could be delivered while minimizing side effects.

There are various elements in a typical DBS signal which can be altered to induce different responses in the brain including: frequency, amplitude, and pulse-on time [173]. This means that any control system utilizing DBS as the effector component has many potential output waveforms, consequently the control systems available for consideration can have significantly more complexity than on-off keying. A number of closed-loop control systems for neurological conditions have been described in the literature which utilize DBS as the effector, including those developed by Bozorgzadeh B, Schuweiler DR, Bobak MJ, et al. [149] and Grahn PJ, Mallory GW, Khurram OU, et al. [157]. The system described in Grahn PJ, Mallory GW, Khurram OU, et al. [157] customizes the activation, amplitude, pulse width, and frequency of the DBS signal based on the output of the control algorithm. There are several DBS devices presented in the literature which could be adapted for use as an effector in an electronic control system, some of these are presented in Table 2.

**Table 2:** DBS devices described in the literature which could be integrated into a control system

Title	Size	Type	Channels	Stimulation Parameters	Battery Life
A Low Power Micro Deep Brain Stimulation Device for Murine Preclinical Research. [203]	13.6 × 10.8 mm	Monophasic	1	Adjustable	12 days
An inexpensive, charge-balanced rodent deep brain stimulation device: a step-by-step guide to its procurement and construction [204]	–	Biphasic	2	Fixed	21 days
Wireless implantable micro-stimulation device for high frequency bilateral deep brain stimulation in freely moving mice [174]	30 × 8 mm	Biphasic	2	Fixed	10 h
A long-lasting wireless stimulator for small mammals [205]	15x8x4mm	Monophasic	1	Adjustable	21 days
Continuous high-frequency stimulation in freely moving rats: Development of an implantable microstimulation system [206]	38x20x13.5 mm	Biphasic	2	Fixed	22 days
SaBer DBS: a fully programmable, rechargeable, bilateral, charge-balanced preclinical microstimulator for long-term neural stimulation [178]	33x20x8mm	Biphasic	2	Adjustable	10 days

The DBS systems presented in Table 2 are all low-cost research devices which would be highly suited for integration into an electronic control system. Almost all of the devices investigated were extremely low power and able to actively produce stimulation signals for multiple days. The device by de Haas R, Struikmans R, van der Plasse G, et al. [174] had the shortest running time, however, this device was entirely implantable and was thus restricted in the size of batteries selected. A number of the devices were only able to produce a fixed stimulation parameter without significantly altering the components included on the circuit board. These devices limit the output options available to an electric control system through altering the amplitude, frequency or pulse width of the supplied stimulation, those devices which can adjust these parameters would be more suited to use in an electronic control system.

A number of the devices examined include multiple channels of stimulation. This is most likely due to the number of studies investigating bilateral DBS which requires a second stimulation channel to be running simultaneously [175–177]. The only device which allows for fully adjustable stimulation of multiple channels simultaneously, is the SaBer DBS device created by Ewing SG, Porr B, Riddell J, et al. [178]. From this review it is clear that there are devices available to provide the stimulation required as a DBS effector in a neurological control system, unfortunately, the literature did not identify any existing systems utilizing mitochondrial function in concert with DBS for potentially increasing the effectiveness of treatment.

### Pharmacotherapy

While DBS is one choice for an effector mechanism it not the only potential candidate, other more traditional pharmaceutical treatment options are also potential solutions. As we develop our understanding of the mechanisms of neuromodulation, we will also see a simultaneous rise in improved technologies for closed loop pharmacotherapy. Closed-loop pharmacology has shown significant benefits in treatments of other disorders such as diabetes and AIDS as well as for administering anesthesia [179–181]. These studies found that administering drugs through closed-loop mechanism could increase precision while attempting to maintain a specific concentration of the drug, while at the same time minimizing the amount of overshoot [180]. A comparative mathematical study performed by Caetano MA and Yoneyama T [181] into closed-loop vs open-loop systems of drug treatment for AIDS found that drug delivery schemes with a closed-loop component improved quality of the treatment, in particular in regards to the quantity of the drugs administered. However, they noted that as the sensor component they were using required frequent and periodic laboratory testing, the closed-loop scheme was inconvenient. If a portable sensor which doesn’t require a laboratory to gather results like those described in Section V were to be used, the potential therapeutic benefits of a closed-loop pharmacological system could be considerable.

A number of studies have found positive results in neurological disorders through pharmacological treatments which target aspects of the mitochondrial respiratory chain [73, 182–186]. A recent study by Karuppagounder S, Madathil S, Pandey M, et al. [184] found that by administering a ROS scavenging flavonoid (quercetin) to rat models of PD, stimulated mitochondrial function and addressed oxidative damage, indicating potential as a neuroprotective treatment for neurodegenerative disease. This result is corroborated by a study conducted by Kaariainen TM, Piltonen M, Ossola B, et al. [187] on the same flavonoid using a different model of PD, however, this second study found that controlling the amount of quercetin administered was of paramount importance as the effects became damaging as the dosage increased. Mitochondrial modulation using nutraceuticals boosting biogenesis is also being explored as a potential treatment for bipolar depression and chronic fatigue syndrome [188]. To support this, technologies must be developed to support safe, precise, and controlled drug delivery.

One solution which shows significant promise is electronically controlled drug infusion micropumps. These micropumps allow for precise control of both the timing and volume of drug delivery [189]. This technology, when integrated with an electronic control system, allows for the realization of dynamic, personalized drug treatment regimens, which are unique to each individual. This maximizes therapeutic outcomes while minimizing side effects and unnecessary treatments [190]. A number of potential drug delivery micropumps are compared in Table 3.

**Table 3:** Comparison of drug delivery micropump devices

Title	Mechanism	Size (mm)	Flow Rate (μl/min)	Pressure (kPa)	Frequency (kHz)	Voltage (V)
A bidirectional silicon micropump [192]	Electrostatic	7x7x2	850	0.31	2	200
A wireless implantable micropump for chronic drug infusion against cancer [194]	Electrolysis	20x15x7.1	2.66	0.69	–	3
Design and test of a high-performance piezoelectric micropump for drug delivery [207]	Piezoelectric	–	3.5 × 10^3^	27	3	50
An Electromagnetically-Actuated All-PDMS Valveless Micropump for Drug Delivery [208]	Electromagnetic	20x12x3.5	319.6	0.95	36.9 × 10^− 3^	–
A self-priming, roller-free, miniature, peristaltic pump operable with a single, reciprocating actuator [209]	Peristaltic	8x22x35	780	48	–	3
A low cost, high performance insulin delivery system based on PZT actuation [210]	Piezoelectric	22 × 18 × 6	4.34 × 10^3^	14.64	0.2	36
An Ultrasonically Powered Micropump for On-Demand In-Situ Drug Delivery [211]	Piezoelectric	22x7x5	13.8	4	–	–
Characteristic studies of the piezoelectrically actuated micropump with check valve [212]	Piezoelectric	10x10x1	1.82 × 10^3^	32	160	120
Design and simulation of a novel electrostatic peristaltic micromachined pump for drug delivery applications [193]	Electrostatic	7x4x1	9.1	–	50	18.5
Piezoelectric micro-pump with PZT thin film for low consumption microfluidic devices [213]	Piezoelectric	30 × 7.5 × 0.15	3.6	4	1	24
An Implantable MEMS Micropump System for Drug Delivery in Small Animals [195]	Electrolysis	–	34	1.31	–	3
Piezoelectric Micropump with Nanoliter Per Minute Flow for Drug Delivery Systems [214]	Piezoelectric	12x6x0.5	4.98 × 10^−3^	–	0.67	16
Design and simulation of an implantable medical drug delivery system using microelectromechanical systems technology [215]	Peristaltic	70x35x1	–	–	–	100
A PMMA valveless micropump using electromagnetic actuation [216]	Electromagnetic	–	400	1.2	12 × 10^−3^	–
A low voltage silicon micro-pump based on piezoelectric thin films [191]	Piezoelectric	30 × 7.5 × 0.15	3.5	3.2	1	24
Study on a piezoelectric micropump for the controlled drug delivery system [217]	Piezoelectric	–	52	–	0.4	140

As can be seen in Table 3, there has been a significant body of research in this area in the past 20 years, with a number of different pumping mechanisms found to be effective. In addition, there are several commercially available options, however, these are resistant to integration with a custom developed control system and thus are not considered. The majority of the pumps examined used a piezoelectric diaphragm based mechanism to move the fluid from the inlet to the outlet, this is possibly due to the simplicity of this mechanism, offering potential for miniaturization. However, these pumps typically require a high voltage to operate, as shown in Table 3, where even the low voltage piezoelectric pump produced by Cazorla P-H, Fuchs O, Cochet M, et al. [191] still requires a voltage of 24 V in order to operate. This is an issue because a typical microcontroller based control system runs at less than 12 V, and often at as little as 3 V DC. This is also an issue in the electrostatic pumps which require up to 200 V AC to operate [192]. The advantage of the electrostatic pumps is their very small size. Both pumps measure less than 49mm^3^ and are among the smallest controllable pumps found in the literature [192, 193]. The advantage of micropumps over other drug delivery solution is the possibility of interfacing them with replaceable reservoirs which allow for long-term treatments to be carried out without replacing the pump.

The pumps developed by Cobo A, Sheybani R, Tu H, et al. [194] and Gensler H, Sheybani R, Li P-Y, et al. [195] appear to be the most suitable for integration into a control system. Both systems run on 3 V DC and have undergone extended testing to verify their precision and performance and would be suitable selections for control by the system outlined in Section VII. Overall, there is a gap of studies that investigate that potential for controllable infusion micropumps to deliver drug treatments for individuals with neurological conditions.

## Discussion

In this paper, we reviewed the literature in order to present an overview of the link between mitochondrial dysfunction and various neurological disorders. Subsequently a generic model of a closed-loop control system for controlling neurological conditions through detecting mitochondrial dysfunction was presented, and the components of the system were examined.

The fact that mitochondrial dysfunction is intrinsically linked to a number of neurological and psychiatric disorders was made clear through the literature, although we note that care must be taken to avoid the “post hoc ergo propter hoc” logical fallacy. Although the disorder and mitochondrial dysfunction are associated, more research is required to determine if mitochondrial function is upstream or downstream, cause or consequence of a disorder. However, in all the disorders reviewed, the mitochondrial function had been identified as a target for future interventions, and in cases where therapies directly targeted mitochondrial dysfunction, neuroprotective outcomes were reported [49, 66, 72, 87]. This is a new area of investigation for some of neurological and psychiatric disorders, which could provide significant therapeutic benefits and greatly improve the quality of life of the great number of individuals with these disorders.

This new line of enquiry could be enhanced through integration with other modern innovations in the biomedical sciences, such as miniaturized closed-loop control systems. The benefits of a closed-loop system assessing mitochondrial function of a patient are self-evident, the treatment a patient is receiving can be altered directly based on the sensed severity of the disorder. This could even be completed on a long-term basis such as the patient being assessed monthly using a non-invasive technique to determine treatment requirements. However, in disorders such as BPD with highly dynamic and symptomatic changes in mitochondrial function, a rapid closed loop system could prove particularly advantageous. In this case, a method of constantly sensing rapid mitochondrial danger signals would have significant potential as a fast responding treatment method which could respond to current environmental demands or to alter based on longer-term state measures of capacity. This type of fast-responding system was investigated further in the generic model presented in this paper.

This model describes a technological solution which is capable of treating these disorders in a manner which integrates feedback, in order to enhance the precision of the treatment delivery. Closed-loop control systems can be personalized to each user, to ensure that the treatment provided is necessary and pertinent, these systems are designed to effectively treat disorders while minimizing wasted treatments [181]. The model provided in Section IV describes the control system as a series of functional components including: the sensor, signal manipulator, controller, and effector. Splitting the model into functional components allows for the discussion of a number of potential options for each component separately.

The first component to be examined was the sensor. A number of sensor technologies were examined including chemiluminescence, amperometry, and fast-scan cyclic voltammetry. By examining these three sensor technologies it is clear that while there are a number of options for determining extracellular ATP concentration and mitochondrial function in-vivo, each of these technologies have distinct advantages and disadvantages. Chemiluminescence is a highly effective sensor technique with a high degree of accuracy, and sensitivity. However, it requires relatively large, sophisticated supporting technology in order to interpret the output from the sensors. In situations where this technology is available and there is sufficient space, this method can be highly effective, however, for a portable long-term closed-loop system this technique is unsuitable. The second technique reviewed was amperometric biosensing. Amperometric biosensing is another highly sensitive technique which avoids the drawbacks found with chemiluminescence, due to the output of the sensor being a comparatively simple electrical signal. However, these sensors take an extended period of time to complete a measurement, which makes them unsuitable for detecting highly transient phenomena and have a finite life. FSCV is a potential solution to this issue as this technique includes the advantages of amperometric biosensing whilst also being able to take measurements with millisecond resolution. In addition, FSCV uses smaller electrodes and does not require bound biological agents on the electrodes in order to take a measurement. Unfortunately, there was no significant literature found which utilized FSCV to sense mitochondrial danger signals, so this technique is as yet unverified. From this, it is obvious that there is no existing ideal solution for determining real-time mitochondrial function in-vivo, and that this would be a valuable area for future engineering researchers to focus on.

The key advantage of the ATP target over existing biomarkers for closed-loop therapies is the focus on measuring underlying causes instead of symptoms. Existing systems utilizing kinematics as a sensing agent for disorder are limited to treating the disorder once it becomes symptomatic rather than much earlier, like in the neural recording system described in Section IV [196]. Another advantage is the selectivity which is available temporally, specially, and chemically, while maintaining the ability to be miniaturized. This type of sensor adds another technique to the neural-sensing toolkit. It could be used in concert with other methods of in-brain sensing, such as local field potentials, and gather information about neural activity as well as ATP movements within the extracellular space [197]. These techniques allow for measurements to be taken at regions, networks and pathways which do not produce local field potentials, giving us a more holistic view of the changes in the brain during disorder.

The review of the controller component was disadvantaged by the fact that the authors could not find any mitochondrial function based closed-loop control system in the literature. In light of this absence, a short review was conducted on a number of similar systems using FSCV techniques to control other neurotransmitters, mainly dopamine. Even with these systems, there was not an extensive amount of literature investigating control schemes. A simple on-off keying control scheme was outlined in the research conducted by Bozorgzadeh B, Schuweiler DR, Bobak MJ, et al. [149]. This type of system is a basic threshold control system, where the output is activated at a particular level and when the measurand reaches a prescribed level the output is deactivated. On the other end of the spectrum was the advanced control scheme used in the Mayo Clinic device described by Grahn PJ, Mallory GW, Khurram OU, et al. [157]. This review did not uncover any published research on the efficacy of different control schemes for control of neurological conditions using FSCV sensors. This lack of research may be explained by the absence of a standard closed-loop platform for testing these schemes. The development of such a platform would be of great benefit when attempting to determine the most effective scheme for treating neurological conditions in this manner.

The final functional component of the generic closed-loop system is the effector. The aim of the effector is to alter the condition of the system in a manner that is adjustable by the controller. In this review, two effector types were considered, DBS devices and pharmacotherapy. There were a number of DBS devices which were suitable for integration into a control system. Due to the relative simplicity of these devices, a number of researchers have developed highly suitable devices which are able to provide DBS pulses in an adjustable and controllable manner. All of the devices surveyed were capable of producing suitable stimulation pulses, and the differentiating factors were other capabilities such as number of stimulation channels, and form-factor. This reflects the mature state of open-loop DBS devices as a whole; the DBS technology is ready for use in closed-loop control systems and a number of systems which use local-field potentials have already demonstrated this fact [198].

The next effector mechanism investigated was micropump systems for controlled delivery of drug therapies. Micropump systems in particular were reviewed due to the ability to be integrated into an electronic control system like that described in Section IV. These systems when integrated with an electronic control system and replaceable reservoirs, allowing for dynamic, highly controlled delivery of drug treatments over extended periods of time. The review clearly showed that there are a large number of suitable candidates for integration into a closed-loop system for neurological conditions. Like the open-loop DBS devices, this reflects the maturity of this technology as an effector mechanism. There were a number of different pumping mechanisms which had been investigated but the electrolysis pumps appear to be the most suitable, as they run on lower voltage levels than other pumping technologies such as piezoelectric. Unfortunately, the authors could not find any published studies in the literature where a drug infusion delivery system had been paired with a neurochemical sensor, for treatment of neurological disorders, which is an area with significant potential benefits. Type one diabetes is an example of a disorder where such a system has been optimized, although the pathophysiology vastly simpler.

## Conclusion

Links between brain mitochondrial dysfunction and neurological and psychiatric disorders are becoming increasingly apparent and represent a targetable neurobiological mechanism for closed loop neuromodulation technologies. A lack of an easy to use, millisecond resolution, in-vivo assaying method is a critical hurdle that must be overcome in order to advance research and therapeutic opportunities in this area. In order to address this issue, we have presented a generic model of a closed-loop control system which utilizes the link between disorders and mitochondrial dysfunction to provide therapeutic intervention, and outlined each of the functional components of such a system. From this investigation, it was clear that there is still a need to carry out further research in this area before a control system such as the one described in the generic model can be developed. The field of low-power closed-loop medical devices is rapidly expanding, and while there are still a number of hurdles to overcome prior to their widespread use, the therapeutic benefits mean that this will be an area for research for a number of years to come.

## Abbreviations

AD: Alzheimer’s disease
ADC: Analogue to digital converter
ADP: Adenosine diphosphate
ALS: Amyotrophic lateral sclerosis
AMP: Adenosine monophosphate
ATP: Adenosine triphosphate
BPD: Bipolar disorder
CCD: Charge-coupled device
CMOS: Complementary metal-oxide-semiconductor
DBS: Deep brain stimulation
DC: Direct current
FSCV: Fast scan cyclic voltammetry
GBD: Global burden of disease
MDD: Major depressive disorder
MOMP: Mitochondrial outer membrane permeabilization
PD: Parkinson’s disease
ROS: Reactive oxygen species
WHO: World Health Organisation
